# Prediction of time averaged wall shear stress distribution in coronary arteries’ bifurcation varying in morphological features via deep learning

**DOI:** 10.3389/fphys.2025.1518732

**Published:** 2025-03-04

**Authors:** Mohammad Hossein Sarkhosh, Hadis Edrisnia, Mohammad Reza Raveshi, Mahkame Sharbatdar

**Affiliations:** ^1^ Faculty of Mechanical Engineering, K. N. Toosi University of Technology, Tehran, Iran; ^2^ Faculty of Mechanical Engineering, Sharif University of Technology, Tehran, Iran; ^3^ Department of Mechanical and Aerospace Engineering, Monash University, Australia

**Keywords:** hemodynamics, deep learning, coronary arteries, bifurcation, computational fluid dynamics (CFD), time-averaged wall shear stress (TAWSS)

## Abstract

**Introduction:**

Understanding the hemodynamics of blood circulation is crucial to reveal the processes contributing to stenosis and atherosclerosis development.

**Method:**

Computational fluid dynamics (CFD) facilitates this understanding by simulating blood flow patterns in coronary arteries. Nevertheless, applying CFD in fast-response scenarios presents challenge due to the high computational costs. To overcome this challenge, we integrate a deep learning (DL) method to improve efficiency and responsiveness. This study presents a DL approach for predicting Time-Averaged Wall Shear Stress (TAWSS) values in coronary arteries’ bifurcation.

**Results:**

To prepare the dataset, 1800 idealized models with varying morphological parameters are created. Afterward, we design a CNN-based U-net architecture to predict TAWSS by the point cloud of the geometries. Moreover, this architecture is implemented using TensorFlow 2.3.0. Our results indicate that the proposed algorithms can generate results in less than one second, showcasing their suitability for applications in terms of computational efficiency.

**Discussion:**

Furthermore, the DL-based predictions demonstrate strong agreement with results from CFD simulations, with a normalized mean absolute error of only 2.53% across various cases.

## 1 Introduction

Despite significant advancements in medical therapy and the availability of both invasive and noninvasive diagnostic tests, cardiovascular disease continues to persist as the leading cause of mortality globally (Maurovich-Horvat et al., 2014; Benvidi and Firoozabadi, 2024). It is widely acknowledged that coronary artery hemodynamics and vessel geometry play a crucial role in the development and diagnosis of coronary artery disease (CAD) (Pinho et al., 2019; Eslami et al., 2020a). Due to the presence of turbulence in the flow, the frictional force exerted on the vessel wall, known as wall shear stress (WSS), can initiate plaque formation at regions of flow separation (Dong et al., 2015; Temov and Sun, 2016).

The left coronary artery and its bifurcations, specifically the left anterior descending (LAD) and left circumflex (LCX), are susceptible to the development of atherosclerosis (Zhang and Dou, 2015; Edrisnia et al., 2024). Additionally, bifurcations pose a heightened risk for restenosis and stent thrombosis (Chaichana et al., 2011; Beier et al., 2016; Freidoonimehr et al., 2021a; Freidoonimehr et al., 2021b). Hence, in clinical practice, it is imperative to assess these regions to identify high-risk patients who may have CAD at an early stage (Hoque et al., 2021). Parameters based on WSS, such as time-averaged wall shear stress (TAWSS) play a fundamental role in the progression of atherosclerosis, particularly in transient studies (Arzani et al., 2021). Studies have shown that a wider bifurcation angle is associated with lower wall shear stress and higher oscillatory shear index (OSI), which can contribute to the stimulation of atherosclerosis (Sun and Chaichana, 2016; Sun and Chaichana, 2017; Liu et al., 2019). Moreover, precise characterization of blood rheology, which involves the application of non-Newtonian models, is particularly crucial in these instances (Pinto and Campos, 2016; Sandeep and Shine, 2021).

Due to the shear thinning behavior of blood in small arteries, CFD is becoming increasingly essential in hemodynamics’ calculations. The accuracy of input data, such as vessel geometry information, appropriate boundary conditions, and material models, significantly influences the reliability and precision of CFD results (Gijsen et al., 2019; Rabbi et al., 2020; Edrisnia and Sharbatdar, 2024). However, the CFD technique is not well-suited for fast-response applications due to the inherent delay and relatively high computational costs involved. As a result, there is a need for an alternative approach to overcome this limitation.

Many state-of-the-art machine learning (ML) techniques, such as Convolutional Neural Networks (CNNs) and Multi-Layer Perceptrons (MLPs), are widely utilized for various applications, including 2D image segmentation and 3D point cloud segmentation (Bello et al., 2020; Hirzi et al., 2022; Sabet et al., 2022). Inspired by these algorithms, there is significant potential for employing them in hemodynamic parameter prediction. However, in scenarios with limited data availability, deep learning models, such as convolutional and recurrent neural networks, often lack robustness and fail to guarantee convergence. Therefore, ensuring access to sufficient and high-quality data is crucial for achieving reliable and accurate results. One promising approach to address data limitations is the use of idealized models in biomedical applications, which can simulate conditions with acceptable agreement to patient-specific cases. These enriched datasets improve algorithm training, enhancing their robustness and performance in real-world applications.

In fluid dynamics applications involving direct calculation of the flow field, ML, and deep learning (DL) approaches have been employed to address computational challenges. These techniques offer promising solutions for expediting computations and enabling fast-response simulations. For instance, Jordanski et al. (Jordanski et al., 2018) have utilized ML techniques to predict the WSS distribution in carotid bifurcation and abdominal aortic aneurysms. Liang et al. (Liang et al., 2017) have deployed a ML approach to establish the connections between shape features and the rupture risk of ascending aortic aneurysms as predicted by finite element analysis (FEA). Furthermore, Liang et al. (Liang et al., 2018; 2020) have developed a DL model, including deep neural networks, to directly estimate the stress distributions in the aorta. Madani et al. (2018) have created data-efficient DL classifiers for cardiology prediction tasks, with a particular emphasis on relevant structures using pipeline-supervised models. Additionally, Madani et al. (2018) have investigated the connection between ML and finite element modeling for WSS prediction of arterial in the context of atherosclerosis. Farnoud et al. (2023) have employed random forest and gradient boosting models for ML analysis of drug delivery to nasal epithelium.

Similar studies are summarized in Table 1. The application of ML or DL to predict hemodynamic parameters, such as Fractional Flow Reserve, FFR, and forecast flow field patterns has remained limited (Itu et al. (2016)). However, there has been an implementation of a reduced-order model, which is highly specialized but has limited applicability. In other investigations, many researchers have employed the Point Net architecture to predict hemodynamic parameters in cardiovascular geometries. Li et al. (2021a) have developed a DL approach to predict the hemodynamics of different cerebral aneurysms before and after the insertion of a flow diverter (FD) stent. Moreover, Li et al. (2021b) have created cardiovascular hemodynamic point datasets and a dual sample channel DL network, capable of analyzing and reproducing the relationship between cardiovascular geometry and internal hemodynamics. The PointNet algorithm, originally proposed for hemodynamic prediction, is also tested in this study to compare its performance with the proposed model, as shown in Table 1.

**TABLE 1:** Comparison of ML- and DL-based methods on hemodynamic prediction.

Methodology	Output	Model	Input	Data set	Performance
Proposed model	TAWSS	Coronary artery bifurcation	Fixed number of point cloud	1800	MRE <11.3% NAME <2.5%
Point Net	TAWSS	Coronary artery bifurcation	Fixed number of point cloud	1800	MRE <24% NAME < −
Neural network approach Itu et al. (Itu et al., 2016)	FFR value	Coronary artery	Geometric parameter	12,000	Error = 0.03%
U-net architecture Su et al. (2020)	WSS	Coronary artery	Fixed number of vessel’s centerline coordination	2000	MAE <11.7 NAME <2.5%
Point net Li et al. (2021a)	Pressure, velocity	idealized cerebral aneurysm	Flexible point cloud	500	MRE <13% NAME <6.5%
Point net Wang et al. (2023a)	Pressure, velocity	Clinical geometry with carotid artery stenosis	High resolution point cloud	1,000	MRE <12.5% NAME <7.5%

Studies on PointNet consistently highlight its strength in capturing global geometric variations but reveal its limitations in addressing local variations. To overcome this, researchers often train separate PointNet models for pre-treatment and post-treatment simulations. In contrast, Su et al. (2020) proposed an alternative approach using idealized geometries with varying stenosis diameters and locations, employing multivariable linear regression (MLR), multi-layer perceptrons (MLPs), and convolutional neural networks (CNNs) as substitutes for computational fluid dynamics (CFD) to compute wall shear stress (WSS) during medical examinations. Although previous study (Su et al., 2020) described their input in Table 1 as a “fixed number of vessel’s centerline coordinates,” they effectively utilized point cloud data in a different coordinate system. Specifically, their input features included the centerline coordinates in the XY plane, along with the distance and angle relative to the centerline. Their study focused on a single coronary artery, varying morphological parameters such as vessel diameter, stenosis, and curvature.

To the best of our knowledge, researchers have proposed DL algorithm for predicting the TAWSS of bifurcations. However, no research has investigated DL-driven prediction on the cases varying in diameters and angulations using U-net structure. For this purpose, point cloud datasets are developed based on CFD simulations of ideal coronary bifurcations, including its hemodynamic parameters. Additionally, a CNN-based U-net is developed to generate the point cloud input for predicting TAWSS at each point. Finally, the effectiveness of the DL approach is assessed by evaluating the prediction errors in TAWSS.

## 2 Material and method

### 2.1 Dataset generation: creation of models and CFD simulation

**FIGURE 1:**
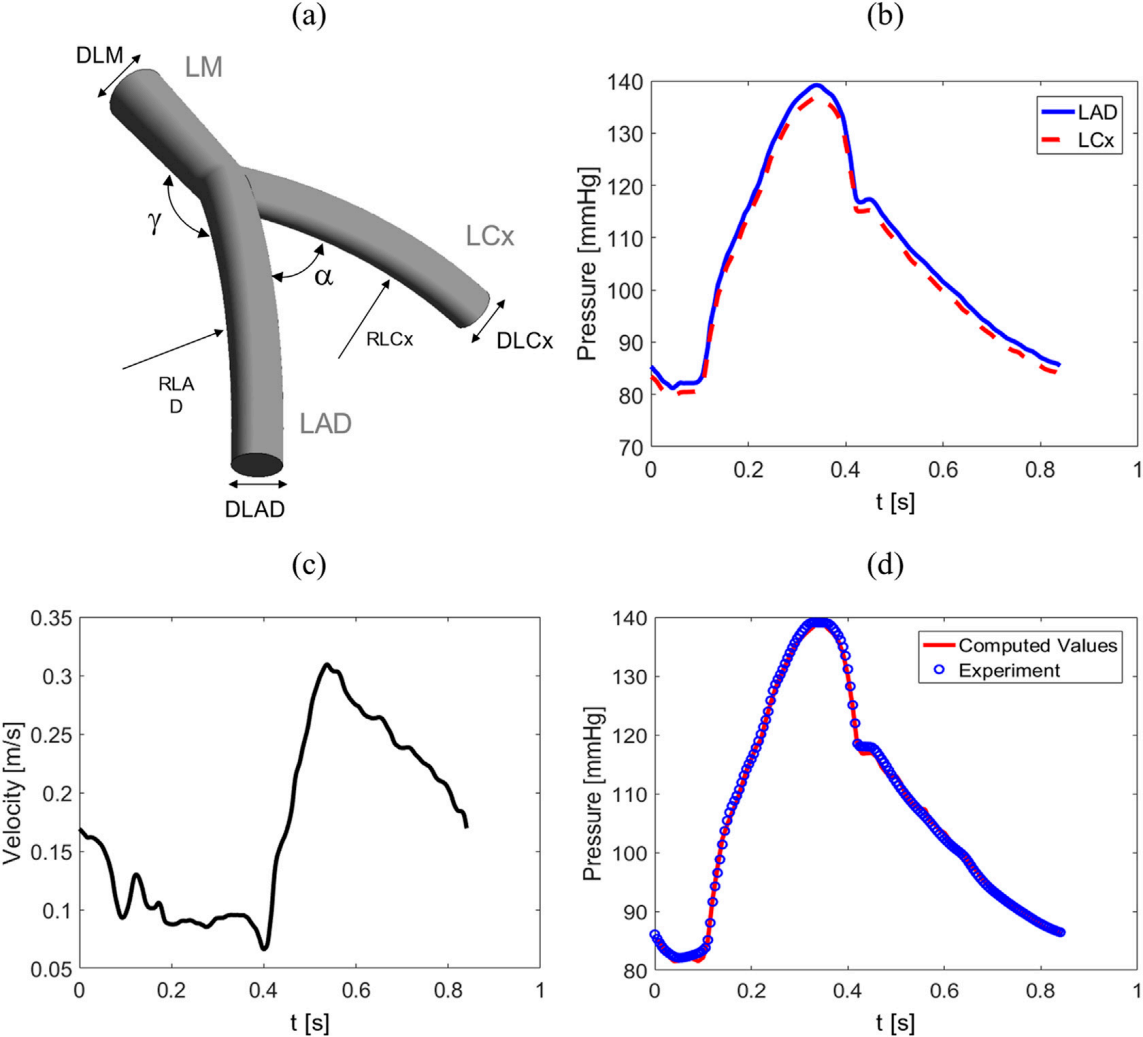
**(A)** An example of an idealized geometry depicting a 3D bifurcation, **(B)** pressure wave for LAD and LCx outlets, **(C)** flow velocity for LM inlet, and **(D)** verification of the results. Comparison between peak inlet pressure at LM in the proposed model and Jahromi et al. study (Jahromi et al., 2019).

To generate the dataset, various healthy coronary artery bifurcation geometries across a broad demographic with transient boundary conditions are simulated using the finite volume method. A 3D parametric model is first established to efficiently create diverse artery geometries as shown in Figure 1A. Furthermore, five morphological parameters, namely, the diameters of the left main coronary artery ( $D_{LM}$ ), left circumflex artery ( $D_{LCx}$ ), and left anterior descending ( $D_{LAD}$ ), along with the angles between LM and LAD (ɣ) and between LAD and LCx (α), are selected. A total of 1800 bifurcation models were generated with scripting option in ANSYS workbench using values selected from a reasonable range, with detailed parameter values shown in Table 2 and corresponding references provided in Supplementary Tables S3, 4. Additionally, Supplementary Figure S2 presents a visualization of a number of generated idealized geometries with varying morphological features.

**TABLE 2:** The range of morphological parameters.

Parameter	$D_{LM}$ (mm)	$D_{LCx}$ (mm)	$D_{LAD}$ (mm)	ɣ (degree)	α (degree)
Range	2.18–4.18	1.5–3.5	1.5–3.5	112.75–172.75	15–130

To create the computational fluid domain, ANSYS meshing software is utilized and a mesh is applied with tetrahedral control volumes in the geometry. Seven prismatic layers with a growth rate of 1.2 are deployed to minimize computational errors near the wall. Moreover, grid independence is studied using the geometry with the largest diameters by evaluating TAWSS values at 6 points on the wall shown in Supplementary Figure S1, across three different mesh resolutions with precise TAWSS values comparison provided in Supplementary Table S2. Therefore, applying this process in a geometry with largest diameters is more effective way than including a larger subset of the 1800 geometries because fine mesh in this case would be extra fine for smaller bifurcation models.

The results of this comparison are presented in Table 3. As depicted, using the medium mesh can provide us with accurate simulations and hence is chosen for its balance between accuracy and computational cost. The difference error with fine mesh, 0.8%, is almost negligible.

**TABLE 3:** Numerical sensitivity study.

Mesh type	Cells number	Node number	Prismatic layer	Error (%)	
Coarse	1,554,000	531,000	7	3.58	
Medium	2,392,000	794,000	7		0.8
Fine	3,342,000	1,085,000	7		

The boundary conditions are representative of a general healthy population obtained from 20 healthy subjects in the Davies et al. study (Davies et al., 2006). They showed that inlet and outlet BCs consist of predominant waves that follow a similar pattern across all subjects during each cardiac cycle. Although the intensity and timing of individual waves varied between the subjects, the wave remained consistently similar in the left coronary arteries. Consequently, this consistency justifies the selection of following conditions for the 1800 geometries studied. For the outlets in the LCx and LAD coronaries are pressure waves throughout a cardiac cycle provided in Figure 1B, while for the inlet in the LM is fully-developed time-varying flow velocity wave shown in Figure 1C. Consequently, considering the mass flow rate, the BCs do not depend on the morphological parameters and mass flow rate can adjust itself in different geometries. To verify CFD models, two diagrams are compared in Figure 1D from the current simulation and data obtained from Jahromi et al. study (Jahromi et al., 2019). The comparison contains the maximum pressure at the inlet in LM.

Each numerical model is extended at inlet to seven times their diameters for fully developed profile and numerical stability, then a flat velocity boundary condition is applied at the inlet (Numata et al., 2017; Su et al., 2020). Furthermore, based on the time step dependency analysis shown in Supplementary Table S1, a time step size of 0.0025 [s] is selected for the simulations. Additionally, the simulations are run over three cardiac cycles to ensure repetitive steady state, as the difference in TAWSS values at the points shown in Supplementary Figure S2 between the third and fourth cycles is only 0.61%, confirming the convergence and periodic consistency of the results. The continuity and Navier-Stokes equations (Equations 1, 2), respectively) governing the blood flow are solved using the CFD software ANSYS-CFX 20.1 (ANSYS Inc., PA, USA):

$$\nabla .\vec{u}=0$$ (1)

$$\rho \frac{\partial \vec{u}}{\partial t}+\rho \left(\vec{u}.\nabla \right)\vec{u}+\nabla p-\mu ∆\vec{u}=0$$ (2)

where $\vec{u},\rho ,p$ and μ represent fluid velocity vector, density, pressure, and dynamic viscosity, respectively.

In the CFD simulation, a high-resolution advection scheme is employed alongside the second-order backward Euler method for time-stepping, achieving a balance between precision and computational efficiency in transient simulations. Convergence is meticulously managed with up to 500 coefficient loops per time step and an RMS residual target of 1E-5 to ensure both accuracy and reliability of the results.

The blood is considered to be an incompressible, laminar fluid with a density of 1,060 kg $/m^{3}$ and assumed to be a non-Newtonian fluid modeled by the Carreau-Yasuda viscosity model (Equation 3). In this model, μ represents the viscosity, $\overset{´}{\gamma }$ is the shear rate and a is the Yasuda exponent. Additionally, the parameters ${m,\mu }_{0},\mu_{\infty }\;\text{and}\;\lambda_{CY}$ represent the Carreau–Yasuda power law index, zero shear viscosity, infinite shear viscosity and time constant, respectively. These parameters are investigated by Sandeep et al. (Sandeep and Shine, 2021) and depicted in Table 4. The equation is provided below:

$$\mu =\left({\;\mu }_{0}-\mu_{\infty }\right){\left(1+{\left(\lambda_{CY}\;\overset{´}{\gamma }\right)}^{a}\right)}^{\left(m-1\right)/a}+\mu_{\infty }$$ (3)

**TABLE 4:** Parameters of Carreau-Yasuda blood viscosity model.

$\mu_{\infty }$	$\lambda_{CY}$	n	a	${\;\mu }_{0}$
0.0035 Pa.s	1.902 s	0.22	1.25	0.056 Pa.s

TAWSS over a cardiac cycle, is calculated to reach a meaningful conclusion and is extracted from each model to be utilized in the DL algorithm. The formula for computing TAWSS is given by Equation 4 as:

$$TAWSS=\frac{1}{T}\int_{0}^{T}\left|\vec{WSS\left(t\right)}\right|dt$$ (4)

Previous studies show different instant WSS values but similar TAWSS values in rigid and fluid-structure interaction (FSI) models (Zeng et al., 2003; Eslami et al., 2020b). Therefore, the coronary artery is assumed to be rigid during the simulations due to computational efficiency.

### 2.2 DL model

#### 2.2.1 Supervised learning

Supervised learning is a ML approach where a model is trained on labeled input-output pairs to learn patterns and predict outcomes on new, unseen data. The process involves minimizing the error between predicted outputs and ground truth labels using various algorithms such as linear regression, support vector machines, and deep neural networks (Rashidi et al., 2019). As TAWSS is continuous, regression algorithms are utilized to predict TAWSS in bifurcation models. In this section, we introduce the input and output of the DL algorithm, followed by the introduction of U-net architecture, tailored for predicting TAWSS in diverse bifurcation models. For this purpose, out of the 1800 models, 60 models are randomly selected in each batch of 300 models, resulting in a total of 360 models used for testing. Additionally, 10% of the remaining 1440 models are chosen for validation from the training set.

#### 2.2.2 Input and output variables

**FIGURE 2:**
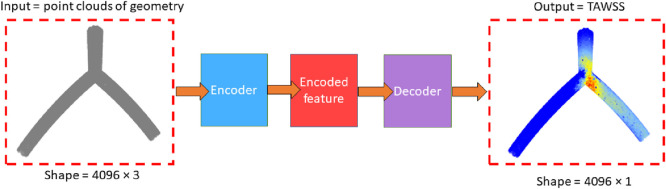
Concept of proposed model.

ML algorithms often struggle with unstructured data. To address this, an interpolation step is applied during post-processing to map the TAWSS values onto a structured surface mesh (4,096 × 3), ensuring that each simulation result is in a structured format. The input of the DL is a point cloud data (mesh nodes coordinates) extracted from CFD-based results with a uniform distribution, representing the geometry of the bifurcation model and the output is the TAWSS at each point. Figure 2 illustrates the structure of the input and output components, designed to replace the function of CFD simulation in fast-response applications. As discussed previously, all geometries have 4,096 nodes, the input includes 3 components of each node ( $x_{i},y_{i},z_{i}$ ), and dimensions of the input and the output are 4,096 × 3 and 4,096 × 1, respectively. The proposed architecture consists of two main parts: encoder and decoder as it is depicted in Figure 2. In the encoder part main features of point clouds are extracted. After which, these features are transformed to the TAWSS values using decoder section.

#### 2.2.3 DL model

U-Net is a CNN structure developed for segmenting biomedical images, specifically for tasks requiring detailed segmentation, it comprises a contracting and expanding path formed a U-shape (Arvind et al., 2023). The contracting path uses convolutional and pooling layers to reduce image dimensions and enhance feature extraction. Meanwhile, the expanding path employs upsampling and concatenation to recover spatial resolution and refine segmentation. Notably, U-Net incorporates skip connections to preserve spatial information, aiding accurate segmentation. Widely adopted, U-Net’s simplicity and adaptability have made it popular in various computer vision applications, including satellite imagery (Alsabhan and Alotaiby, 2022) and road segmentation (Wang R. et al., 2023).

**FIGURE 3:**
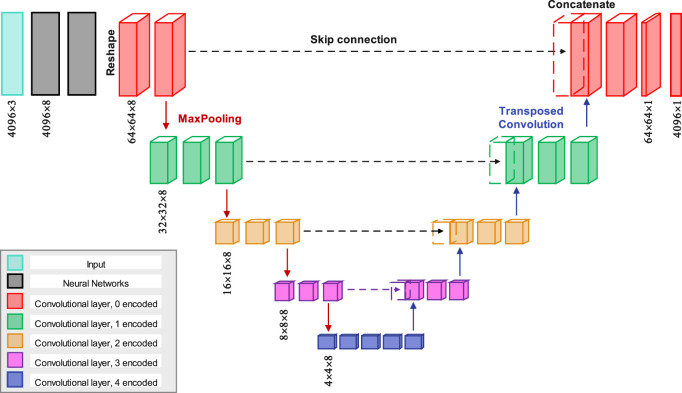
The proposed model features a CNN layer structured in a U-Net architecture, where each encoded and decoded layer is color-coded for clarity. The skip connections transfer encoded data to their corresponding decoded layers for concatenation.

The architecture used to derive TAWSS from the point clouds of geometry is a modified U-net structure, as shown in Figure 3. Initially, the resolution of the input geometry increases from 4,096 × 3 to 4,096 × 8 after two neural network layers. Then it is reshaped to a dimension of 64 × 64 × 8, followed by the application of a convolutional layer with same depth to 64 × 64 × 8. Another convolutional layer is then applied, maintaining the same dimensions. In the encoder section, four max-pooling operations progressively halve the spatial dimensions. For instance, the first max-pooling layer reduces the dimensions from 64 × 64 × 8 to 32 × 32 × 8. After each max-pooling step, two convolutional layers are applied while keeping the same dimensions. Once the final max-pooling is completed, the encoded data passes through four additional convolutional layers.

In the decoder section, four transposed convolution layers are used to progressively restore the spatial dimensions. For example, the first transposed layer expands the dimensions from 4 × 4 × 8 to 8 × 8 × 8, followed by a skip connection that merges the corresponding feature maps from the encoder path. As shown in Figure 3, two additional convolutional layers with dimensions of 8 × 8 × 8 are applied. This process is repeated for the subsequent transposed layers. To mitigate overfitting during training, an initial learning rate of 0.001 is used, with exponential decay applied every 2000 steps. Additionally, batch normalization is implemented after each convolutional layer and before the ReLU activation, except for the output layer, where it is deemed unnecessary for regression operations. Moreover, all convolutional layers utilize a 3 × 3 kernel size.

To quantitatively assess the discrepancy between the outcomes predicted by the DL and CFD simulations, we referenced previous studies (Li et al. (2021a), Li et al. (2021b), Wang et al. (2023a)) and employed the mean radial error (MRE) and normalized mean absolute error (NAME). These metrics were used to evaluate the error magnitude at individual mesh points (Li et al., 2021a; Wang S. et al., 2023). MRE quantifies the difference between the predicted DL values and the corresponding actual values across all points within the model. In contrast, NAME measures the deviation of the DL-derived results from the actual values across the entire flow field represented by CFD results. The precise formulations of MRE and NAME are provided in Equations 5, 6.

$$MRE\left(y,\hat{y}\right)=\frac{1}{N}\sum_{i=1}^{N}\frac{\sqrt{{\left(y_{i}-\hat{y_{i}}\right)}^{2}}}{\sqrt{y_{i}^{2}}}\times 100\%$$ (5)

$$NAME\left(y,\hat{y}\right)=\frac{1}{N}\frac{\sum_{i=1}^{N}\left|y_{i}-\hat{y_{i}}\right|}{\mathrm{Max}\;\left|y\right|-\mathrm{Min}\;\left|y\right|}\times 100\%$$ (6)

where $y_{i}$ and ${\hat{y}}_{i}$ denote the *i*th values of TAWSS obtained by DL-predicted and CFD-simulated results, respectively. i is the point spatial sequence, N is the total number of nodes, || denotes the absolute value. NAME measures the absolute error for each individual data point after normalizing it by the peak value, and then calculates the average. It is essential to emphasize that max{y} represents the highest value obtained from these 1800 models.

To optimize the DL model, an additional study of the network layers is provided in the Supplementary Material. Supplementary Table S5 investigates the effect of encoding/decoding sessions, revealing that increasing the number of sessions enhances prediction performance. Supplementary Table S6 demonstrates that utilizing four convolutional layers in the final encoding section yields the best prediction accuracy. Additionally, Supplementary Table S7 highlights the significant impact of skip connections, showing that they can reduce the MRE by half, further improving prediction performance.

This investigation is carried out on a Windows 10 workstation featuring a 3.4 GHz CPU and 128 GB of RAM. CFD simulations are performed using ANSYS-CFX 20.1 (ANSYS Inc., PA, USA). The computational time varies, usually below the 50 minutes for most cases, with an average duration of approximately 45 min. We implement the DL models using TensorFlow 2.0 and expedited the training process with a 12G TITAN Xp GPU. The training phase for the proposed model, with 27,339 parameters, lasted approximately 2 hours. The dataset preparation, which involves simulating 1800 idealized models, required about 1350 h. Nevertheless, all neural networks demonstrated the ability to generate a TAWSS map in less than 1 s during testing.

## 3 Results

Ordering point clouds is crucial, particularly in CNN architectures, as it helps extract local features. Principal Component Analysis (PCA) ordering is used which is one of the simplest methods for imposing order. This approach aligns the point cloud with its principal axes by computing eigenvectors of the covariance matrix. The points are then sorted based on their projections along the principal eigenvector, which corresponds to the direction of maximum variance. To assess its functionality, this ordering technique is compared with x, y, and z ordering points. As it is concluded from the Table 5, the MRE and NAME for ordering in canonical order is much better, 11.3 and 2.5, respectively.

**TABLE 5:** Result of training the proposed U-net with ordering points in x, y, z-directions, and PCA ordering.

Order	MRE	NAME
x-ordering	34 ± 8.3	3 ± 1.2
y-ordering	29.1 ± 7.5	3.6 ± 1.1
z-ordering	27.6 ± 3	3.7 ± 1.8
Principle axes ordering	11.3 ± 5.5	2.5 ± 0.6

As shown in Figure 3, one of the steps in the process involves reshaping 4,096 points into a 64 × 64 format. Specifically, the points are grouped into 64 clusters, each containing 64 points. After applying ordering to the point clouds, the grouping is schematically illustrated in Supplementary Figure S3.

**FIGURE 4:**
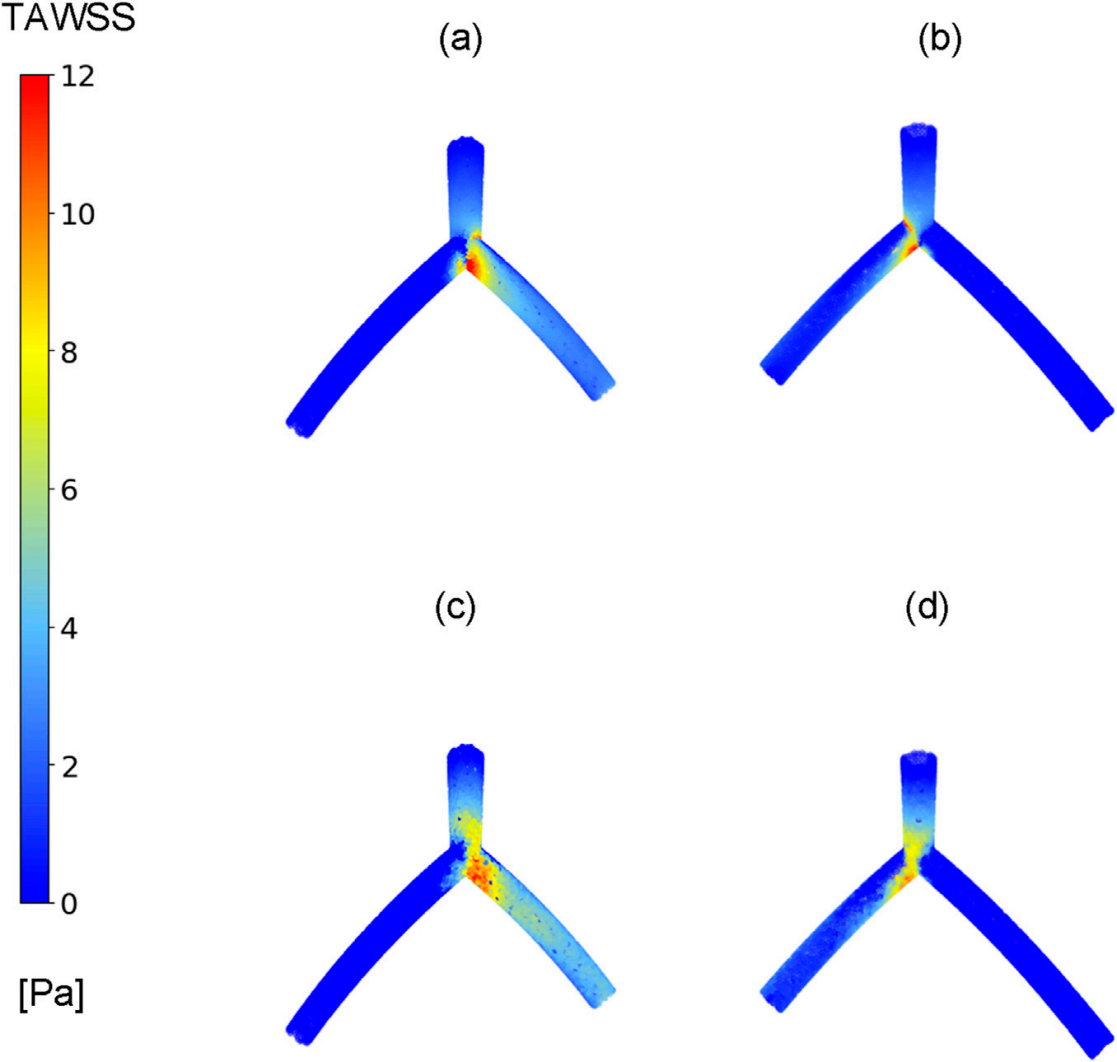
TAWSS distribution. Comparison between CFD results **(A, B)** and proposed DL model results **(C, D)** in one case in two different views, **(A, C)** TAWSS contours in front view, **(B, D)** TAWSS contours in back view.

In this study, our primary focus is on examining the hemodynamic parameter known as TAWSS, a metric previously investigated in studies related to coronary arteries (Eshtehardi et al., 2012; Su et al., 2020). Figure 4 compares the TAWSS values obtained from the CFD and DL models across two perspectives. Due to the curved geometry of the LAD and LCx in the idealized model, the TAWSS distribution is asymmetrical between the front and back views. Notably, the TAWSS values in the front view, as shown in Figures 4A, C, are almost 25% higher than those in the back views depicted in Figures 4B, D. In Figure 4A, left and right branches correspond to the LAD and LCx arteries, respectively. Although the values demonstrate good predictive capabilities in the LM and LAD vessels, the exact TAWSS values distribution from the CFD simulation (Figures 4A, B) has higher values in the bifurcation and LCx artery in this case.

**FIGURE 5:**
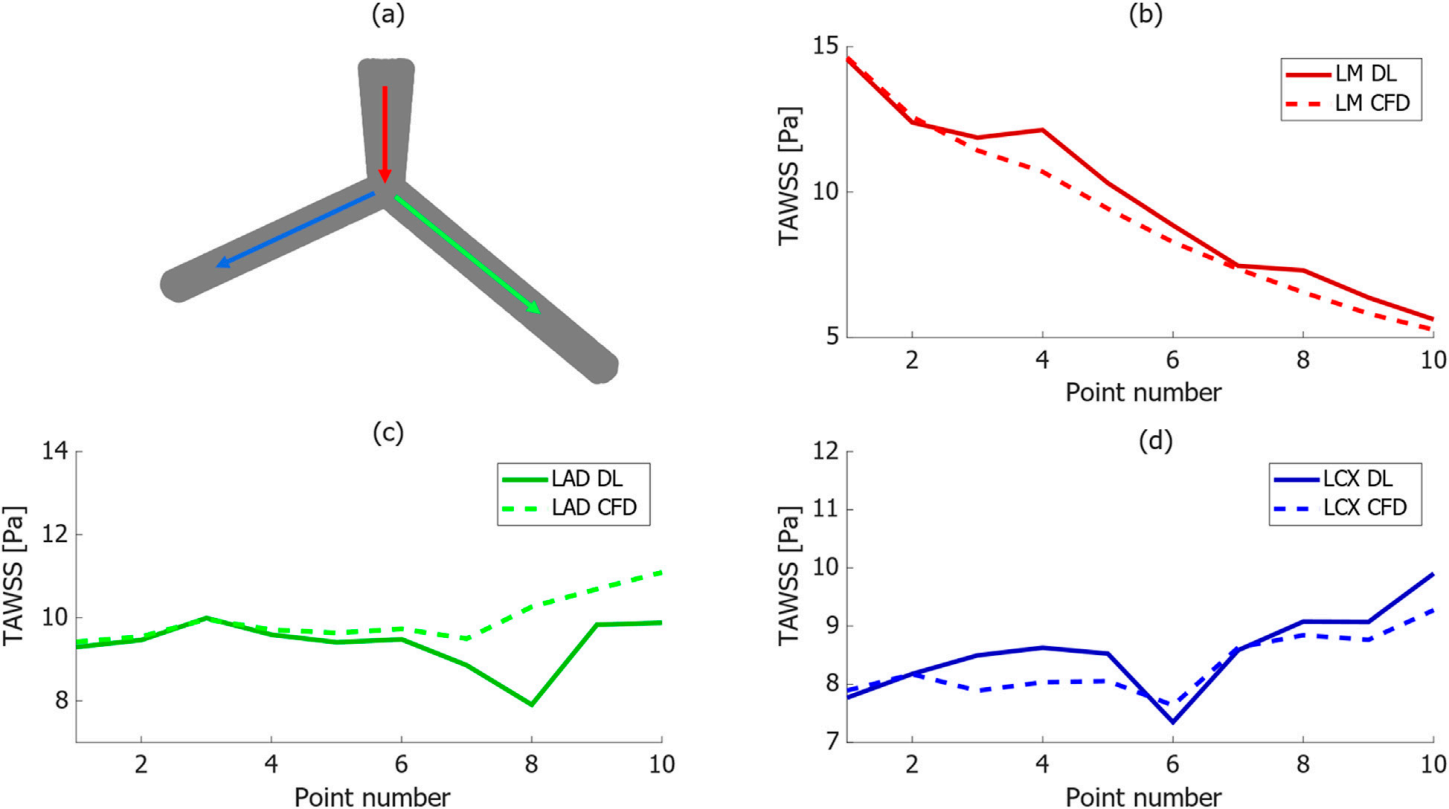
TAWSS distribution comparison between CFD and proposed DL models along three paths in a bifurcation, **(A)** schematic of selected paths along the vessels in the bifurcation geometry, **(B–D)** comparison between TAWSS values in DL and CFD results along the selected paths.

Figure 5 depicts the comparison of TAWSS distribution between CFD and DL models along three lines on the wall within a bifurcation, representing specific paths of interest on the walls of LM, LAD, and LCx arteries. Each line consists of ten points, evenly spaced in the same direction as indicated by the arrow, to report the TAWSS value. This analysis evaluates the DL model’s ability to approximate the TAWSS distribution compared to the CFD approach from simulation. Generally, trends agree quite well in the models, however, in some points the absolute error is higher compared to other regions. For instance, in Figure 5C, it can be observed that in TAWSS graph along the LAD, in one point the difference reaches 20%.

Notably, the TAWSS value line graph for the LM artery in Figure 5B reduces from more than 15 [Pa] to 5 [Pa] at the outlet, with the DL model accurately predicting this trend. Moreover, TAWSS values along the LAD artery in Figure 5C remain relatively constant, fluctuating between 10 [Pa] and less than 12 [Pa] from the CFD and DL models, respectively. Additionally, there was a slight increase in TAWSS values along the line on the wall of LCx artery in the CFD model shown in Figure 5D, from 8 to 10 [Pa], with a less than 0.5 [Pa] difference in the DL model.

**FIGURE 6:**
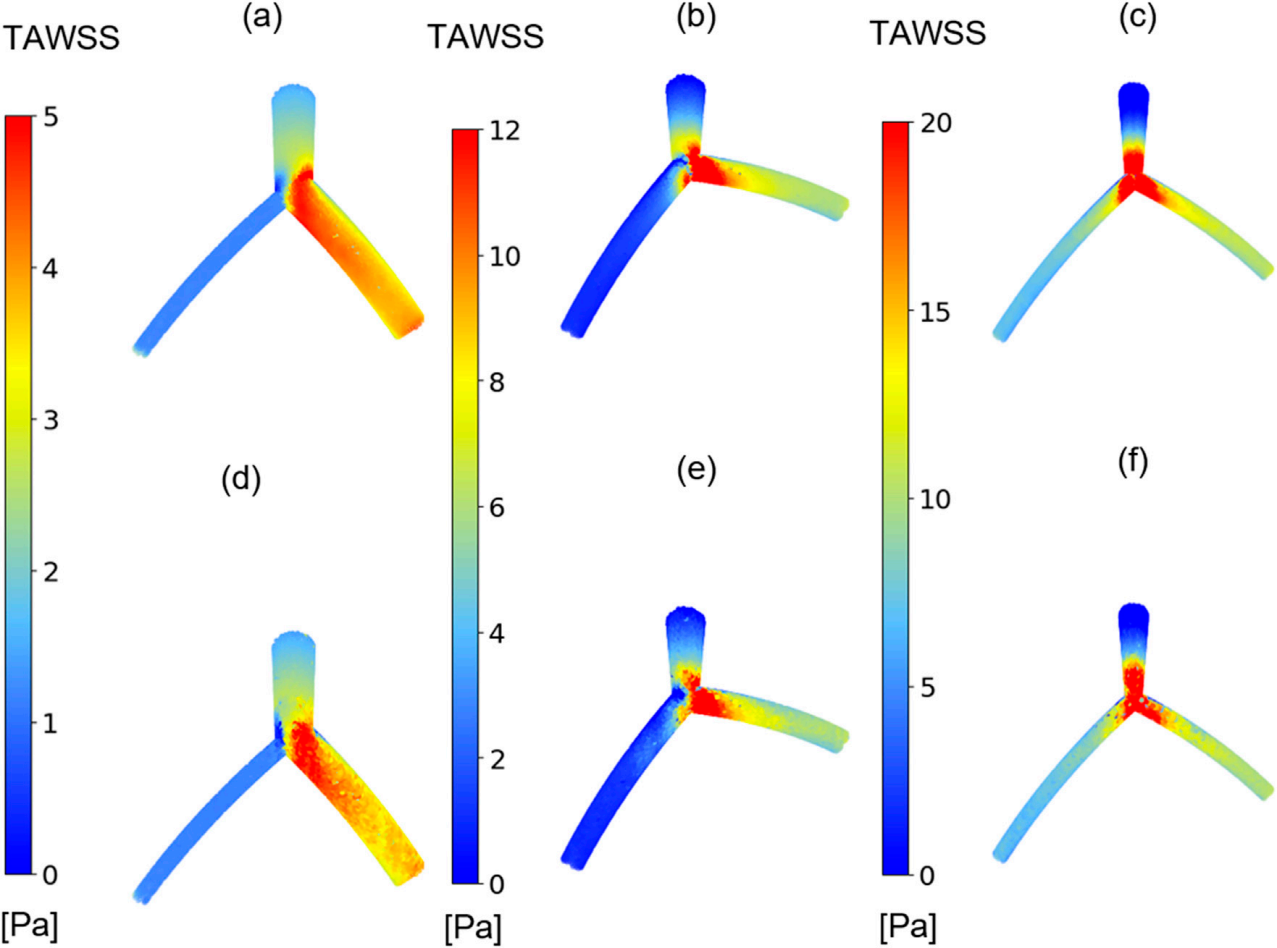
TAWSS distribution. Comparison between TAWSS values from CFD results **(A–C)** and proposed DL model results **(D–F)** in three cases with varying morphological parameters.

Figure 6 presents the results of TAWSS distribution obtained from CFD simulations (depicted in Figures 6A–C and the DL predictions (illustrated in Figures 6D–F, showing three different models with varying morphological parameters and associated errors. In all models, the left and right branches correspond to the LCx and LAD arteries, respectively. In branch region, the DL model tends to underestimate TAWSS values in LCx in Figure 6F). Nevertheless, it is evident that it is capable of capturing the overall pattern and values of TAWSS distribution.

**FIGURE 7:**
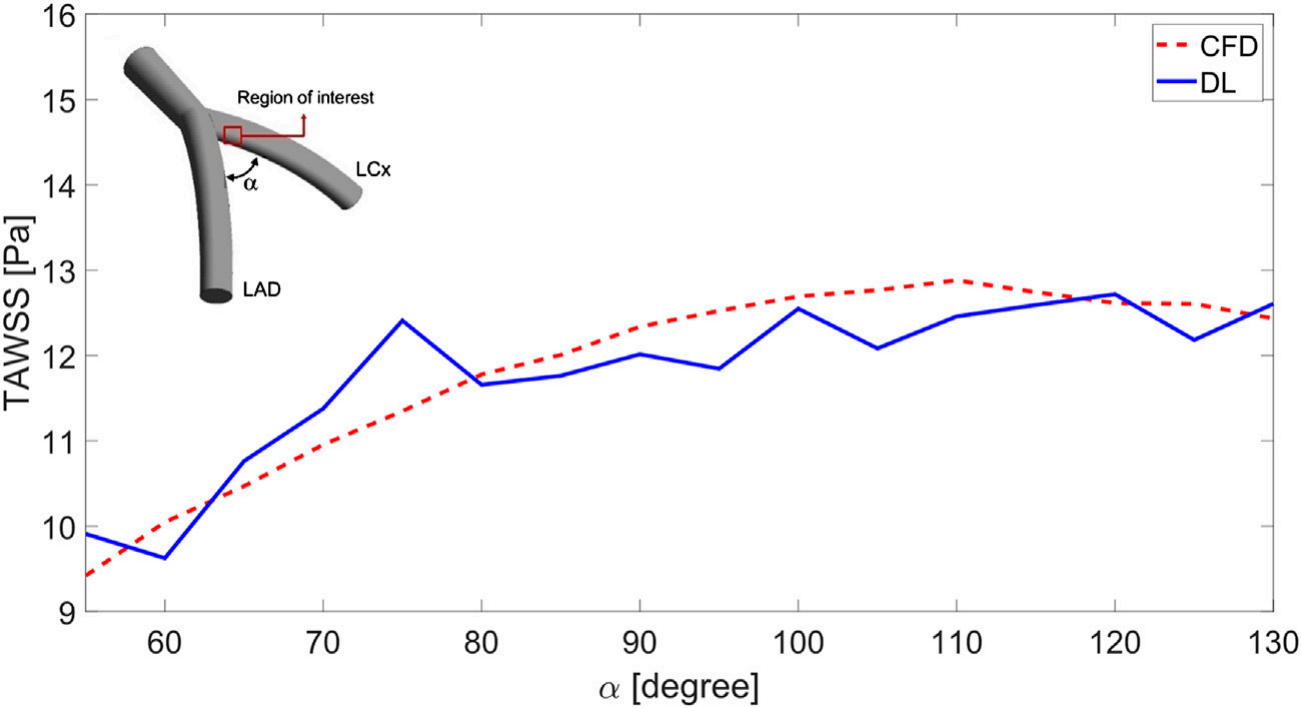
TAWSS prediction in the region of interest varying in the α angle between LCx and LAD arteries.

Figure 7 presents a line graph comparing the TAWSS distribution in a region of interest on the LCx for varying α angles in two models. The TAWSS values are averaged across the region’s points, and the comparison is made between CFD and DL model outputs. The results show that the DL model accurately captures the overall increasing trend of TAWSS as the angle widens. Beyond 110°, TAWSS values plateau, although the DL model introduces minor variations. At smaller angles (below 70°), the differences between the models are minimal, remaining under 1.6 Pa, with the DL model closely matching the rising trend. However, at 75 and 105°, the model shows larger deviations, with discrepancies of 1.5 Pa and 1.1 Pa, respectively. While the DL model generally performs well in predicting TAWSS, fluctuating patterns in DL model suggest that further refinement is needed to improve its accuracy.

## 4 Discussion

Su et al. (2020) achieved real-time 2D WSS prediction by creating numerous idealized blood vessel models derived from a limited clinical dataset and utilizing a CNN. It is shown in this study that CNN works better than MLP and MLR. However, their study does not include more complex geometries like bifurcations. Moreover, in their input they utilized 2D information of geometry which is much more straightforward than using a point cloud as an input. The Point Net structure, on the other hand, provides a significant advantage for geometries before and after treatment. Li et al. (2021a) and Wang et al. (2023b) applied the same structure for idealized geometry with cerebral aneurysm and patient-specific carotid artery geometries with stenosis, respectively. Despite its benefit that is robust to the number of input models, Point Net faces a prominent challenge in terms of variation in geometry features. As in the previous investigation, the Point Net model was trained separately for carotid arteries with and without stenosis (Wang S. et al., 2023).

However, in the proposed model not only the model is trained to predict data varying in the most crucial parameters like diameters and angels of bifurcation, but also it utilizes a point cloud as the input. The error function results for TAWSS at each mesh point, evaluated across the testing set models using the MRE Equation 5 and NAME Equation 6, were 11.39 ± 5.5 and 2.5 ± 0.56, respectively. It is important to note that the model was not previously trained on the testing set, which comprised 360 bifurcation models.

Due to the high dimensions of input data, CNN is a great tool for encoding the point cloud since it reduces trainable parameters rather than using MLP. In CNNs, each neuron in a layer is linked to a small region of the previous layer, known as the receptive field. This sparse connectivity minimizes redundancy, allowing the network to focus on local patterns. In the case study of Gharleghi et al. (Samarasinghe, 2020), they used CNN structure to predict the TAWSS in bifurcations, however, they used unfolding techniques to make 3D point clouds to 2D data. In case of bifurcation there is a complex relation between points in the center of geometry. Unfolding point clouds leads not to consider the relation between points where CNNs solely rely on local data. As a result, this structure cannot predict wide ranges of characteristics.

The proposed DL model for predicting TAWSS in coronary artery bifurcations offers substantial advantages for medical applications, particularly in real-time diagnostics and treatment planning. Traditional CFD simulations, though accurate, are computationally intensive and impractical for fast-response scenarios in clinical settings. Our DL-based approach, which predicts TAWSS with a normalized mean absolute error of 2.53% in under one second, overcomes this challenge by drastically enhancing computational efficiency without compromising accuracy. This advancement facilitates rapid, patient-specific assessments of hemodynamic factors linked to atherosclerosis and stenosis, enabling timely decision-making for both surgical and non-invasive interventions. Additionally, the model’s versatility across various coronary geometries further increases its clinical applicability, presenting a promising tool for personalized cardiovascular care.

## 5 Limitations and future remarks

A limitation we faced was the lack of patient-specific bifurcation models. To address this, we generated artificial bifurcation datasets for DL model, incorporating morphological parameters as substitutes for real-world data. Consequently, the optimal network parameters derived from the training samples deviated from actual clinical scenarios. Furthermore, during the CFD simulation process, we applied generic boundary conditions instead of tailored, patient-specific ones. Nevertheless, this approach is consistent with previous simulation studies (Davies et al., 2006; Jahromi et al., 2019). Another limitation was the use of a fixed number of point cloud as a input of the model, which could vary depending on the complexity of DL networks. Additionally, since our DL model’s input consisted of a uniform and fixed number of point cloud points across the geometries, some transitions in TAWSS values may have been overlooked in certain regions. Despite these constraints, we present a DL method that takes into account various restrictions, including dynamic flow boundary conditions. The DL model achieved an MRE value of 11.39 on the test dataset, successfully capturing the overall trend. However, a point-by-point analysis along the three vessels in Figure 5 reveals that the absolute relative error at one point reaches 20%. This result shows a limitation of the DL model, which could be addressed in future studies. Other parameters, such as velocity and pressure, are vector quantities, requiring predictions in three directions at each point. Additionally, velocity and pressure are defined for the fluid domain, making their complexity different from TAWSS, which is only defined on the wall. These aspects can be explored in future studies.

As a proof of concept, this study demonstrates the feasibility of using a DL-based approach for fast and accurate TAWSS prediction. The model achieved a NAME of only 2.5% and generate predictions in less than one second, underscoring its potential for rapid clinical applications. The efficiency gains, along with the strong correlation to CFD results, represent valuable contributions to the field.

## 6 Conclusion

In this study, we introduced a simulation-based framework for the first time to achieve Unet-based prediction of TAWSS in coronary arteries bifurcation with varied diameters and angels. Through the generation of high-quality point cloud datasets and the utilization of U-net, we demonstrated that the DL-based approach yields highly accurate predictions closely aligning with CFD simulations. Importantly, this achievement is accompanied by a notable reduction in computational costs. This emphasizes the potential and viability of the DL-based strategy for rapid and precise forecasting of TAWSS in coronary arteries bifurcation.

## Data Availability

The original contributions presented in the study are included in the article/Supplementary Material, further inquiries can be directed to the corresponding author.
