# Supplementary material for: Prediction of time averaged wall shear stress distribution in coronary arteries’ bifurcation varying in morphological features via deep learning
Source: Front Physiol. 2025 Mar 4;16:1518732. doi: 10.3389/fphys.2025.1518732 (PMC11920710; doi:10.3389/fphys.2025.1518732)
Supplement: Supplementary file 1 [file Table1.docx]

**Prediction of time-averaged wall shear stress distribution in coronary arteries’ bifurcation varying in morphological features via deep learning**

* Corresponding author, [m.sharbatdar@kntu.ac.ir](mailto:m.sharbatdar@kntu.ac.ir)

Faculty of Mechanical Engineering, K. N. Toosi University of Technology, Tehran, Iran

The PDF file includes:

Figure S 1: Illustration of the six selected points used for the mesh, time step, and cycle independence study.

Figure S 2: Visualization of generated idealized geometries varying in morphological features.

(a) D_LM_:2.18- D_LAD_:1.5- D_LCx_: 1.5- ɣ:159- α:90, (b) D_LM_:4.18- D_LAD_:3.5- D_LCx_: 3.5- ɣ:159- α:90, (c) D_LM_:4.18- D_LAD_:3.5- D_LCx_: 3.5- ɣ:112.75- α:130, (d) D_LM_:4.18- D_LAD_:3.5- D_LCx_: 3.5- ɣ:160- α:45, (e) D_LM_:4.18- D_LAD_:3.5- D_LCx_: 3.5- ɣ:112.75- α:90, (f) D_LM_:4.18- D_LAD_:3.5- D_LCx_: 3.5- ɣ:159- α:120.

Figure S 3: A 2D representation of one of the point clouds after ordering. The 64 clusters, each containing 64 points, are shown in different colors to illustrate the effect of ordering.

Table S 1: Analysis of timestep independency at six different points.

Table S 2: Analysis of mesh independency at six different points.

Table S 3: Bifurcation Angulation geometry – Left Coronary Artery depicted in Figure 1.a.

Table S 4: Arteries’ Diameters– Left Coronary Artery depicted in Figure 1.a.

Table S 5: Comparison of Encoding and Decoding Sessions on Prediction Performance.

Table S 6: Effect of the Number of Convolutional Layers in the Final Encoding Section on Prediction Performance.

Table S 7: Impact of Skip Connections on Prediction Performance.

**SUPLEMENTARY MATERIAL**

**
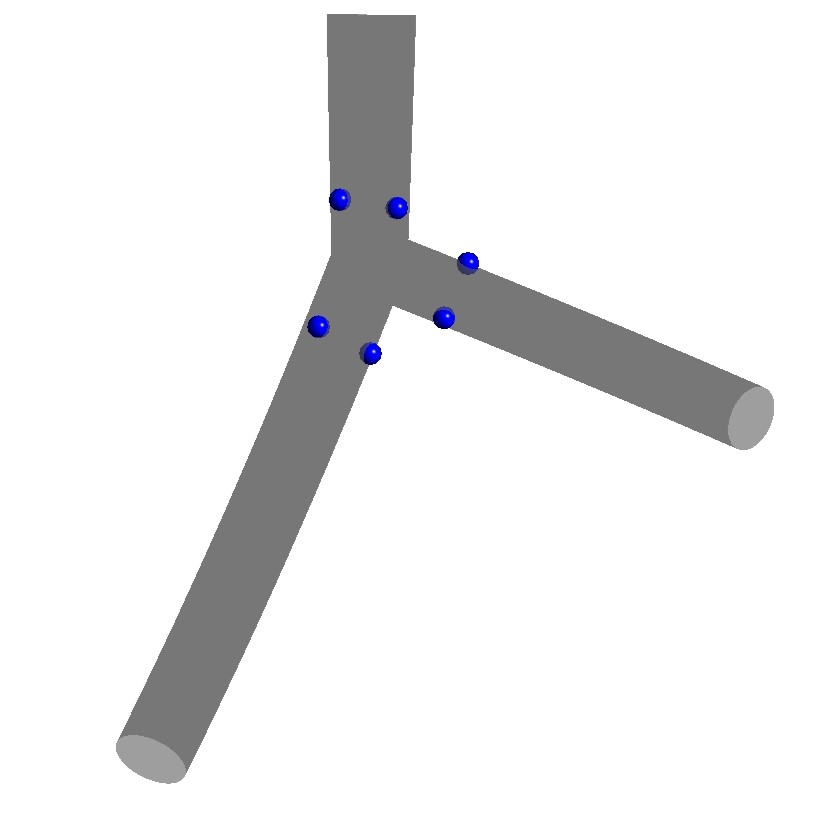
**

Figure S 1: Illustration of the selected points used for the mesh, time step, and cycle independence study.

Table S 1: Analysis of timestep independency at six different points.

|  |  | TAWSS [Pa] | | | | | |
| --- | --- | --- | --- | --- | --- | --- | --- |
| Point  Timestep [s] | | 1 | 2 | 3 | 4 | 5 | 6 |
| 0.02 | | 8.57 | 3.28 | 2.15 | 2.35 | 0.44 | 4.76 |
| 0.01 | | 8.52 | 3.27 | 2.13 | 2.37 | 0.43 | 4.75 |
| 0.005 | | 8.51 | 3.27 | 2.13 | 2.36 | 0.42 | 4.74 |
| 0.0025 | | 8.49 | 3.27 | 2.12 | 2.369 | 0.42 | 4.74 |
| 0.00125 | | 8.49 | 3.27 | 2.12 | 2.37 | 0.42 | 4.74 |

Table S 2: Analysis of mesh independency at six different points.

|  |  | TAWSS [Pa] | | | | | |  |  |
| --- | --- | --- | --- | --- | --- | --- | --- | --- | --- |
| Point  Mesh | | 1 | 2 | 3 | 4 | 5 | 6 | Error% | |
| Coarse | | 9.55 | 2.76 | 2.34 | 2.15 | 0.46 | 4.38 | 2.51 | - |
| Medium | | 9.18 | 2.71 | 2.21 | 2.16 | 0.47 | 4.29 |  | 0.89 |
| Fine | | 9.11 | 2.67 | 2.16 | 2.17 | 0.47 | 4.27 | - |  |

| 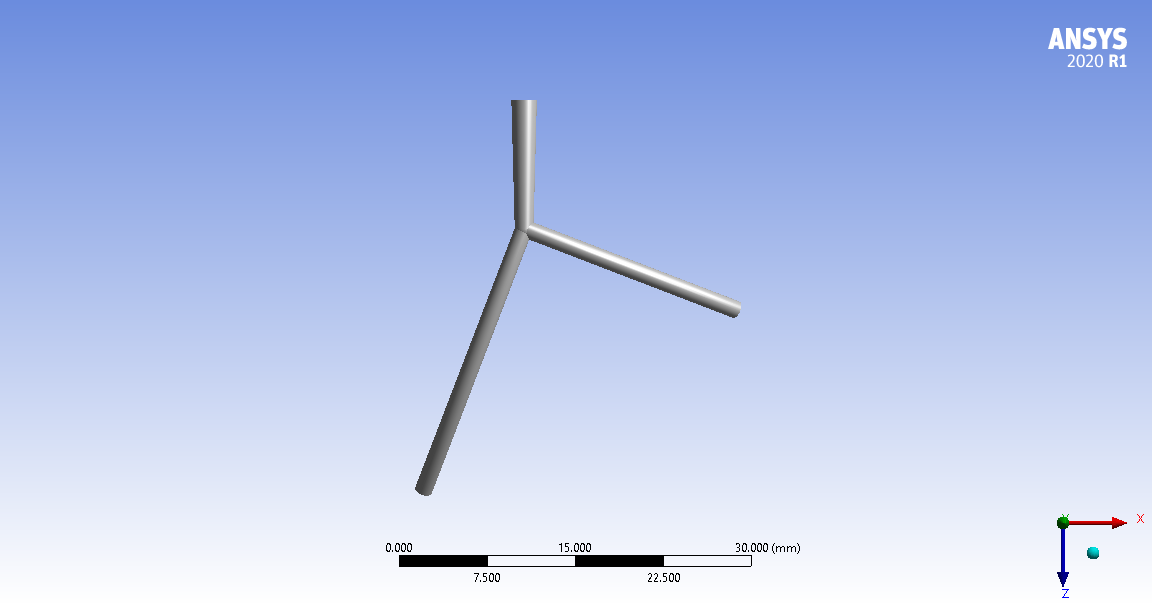 | 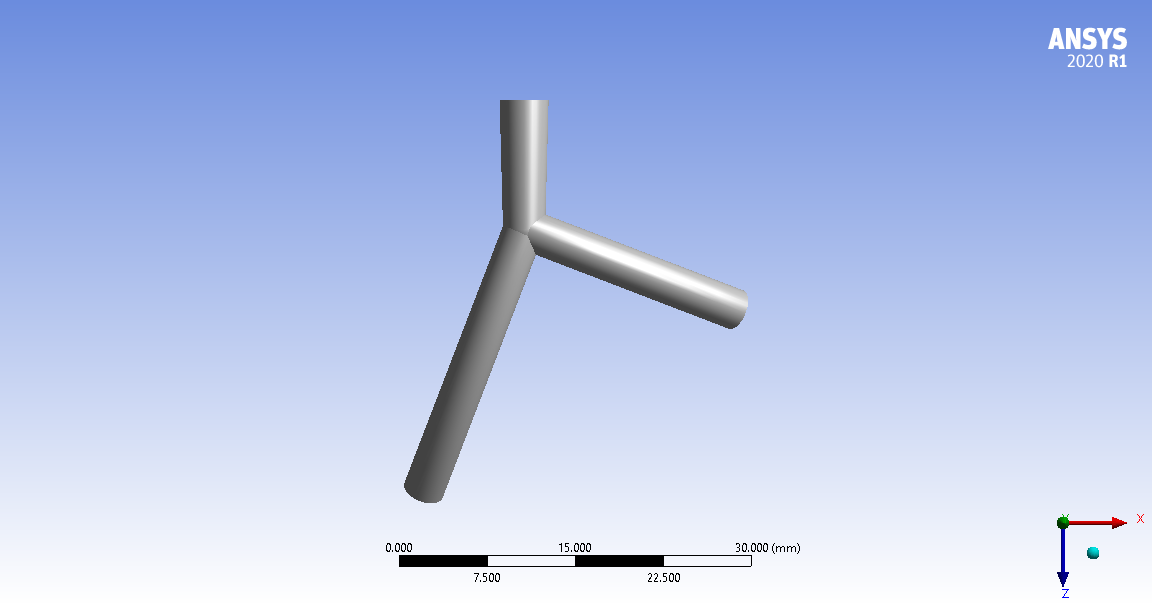 |
| --- | --- |
| (a) | (b) |
| 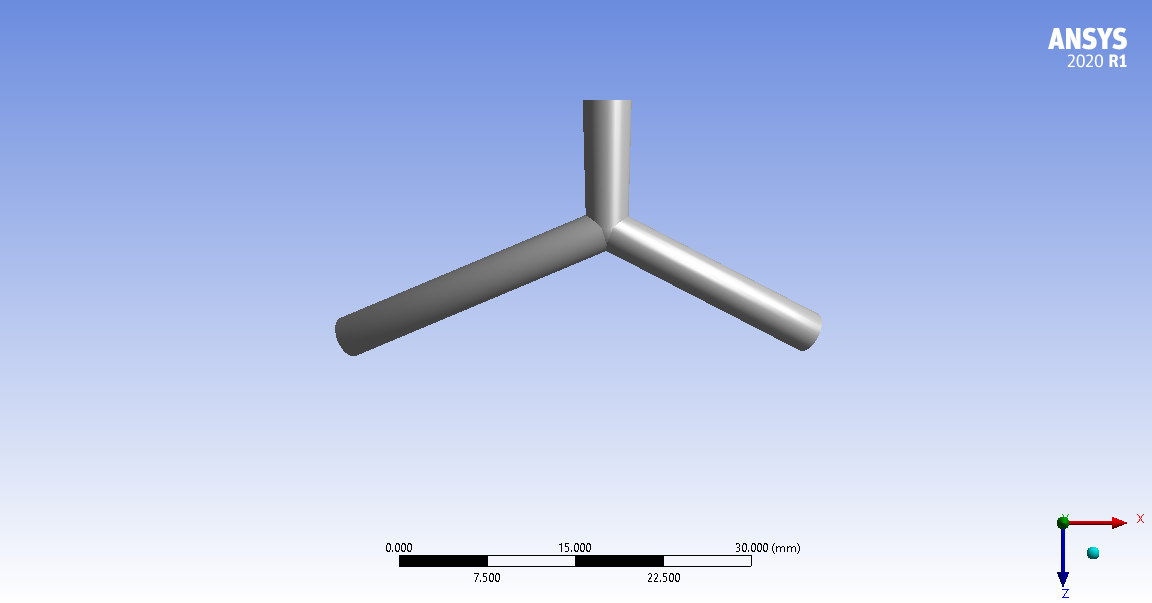 | 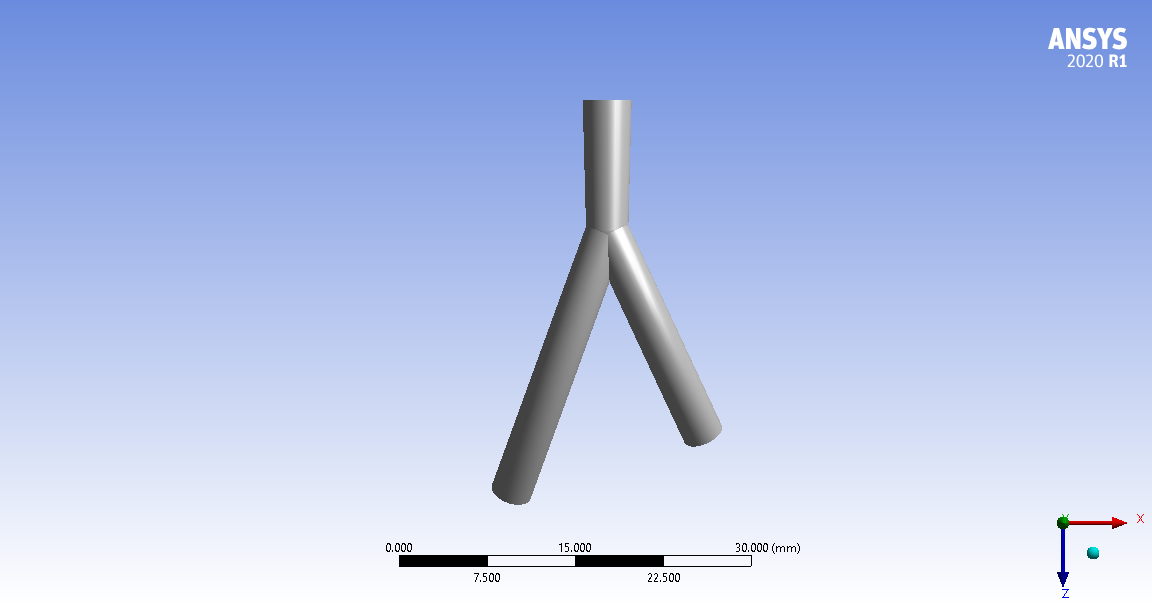 |
| (c) | (d) |
| 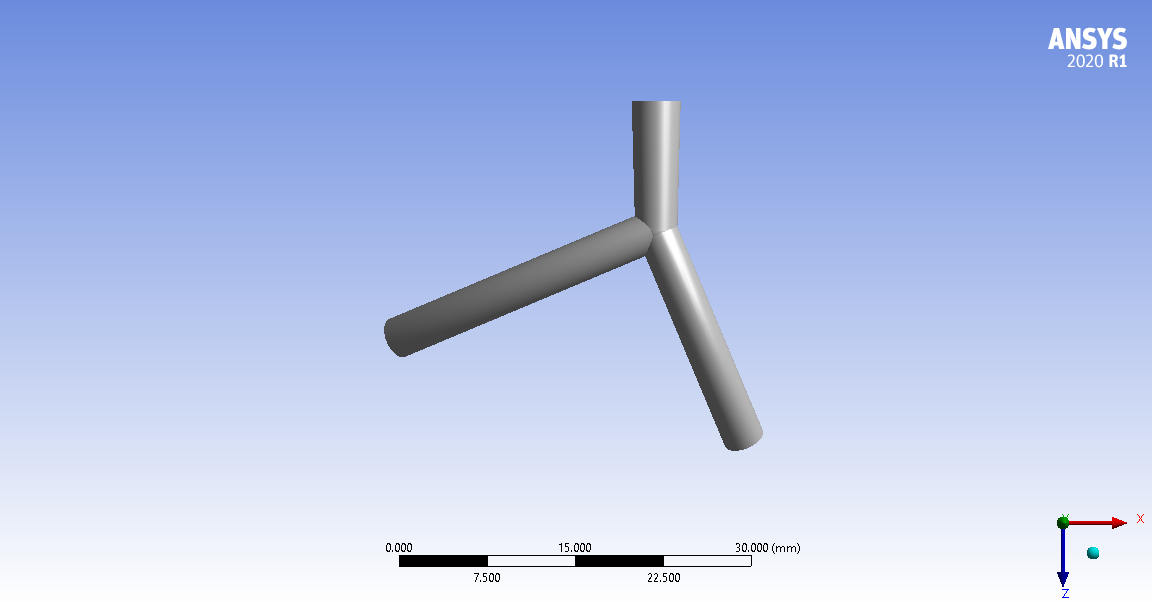 | 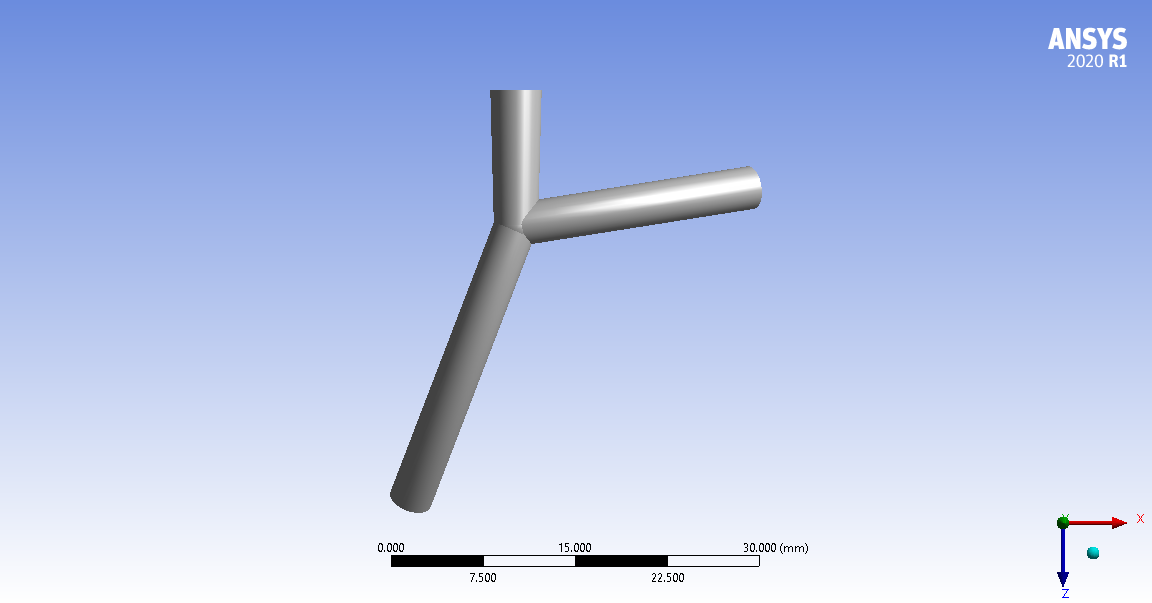 |
| (e) | (f) |

Figure S 2: Visualization of generated idealized geometries varying in morphological features.

(a) D_LM_:2.18- D_LAD_:1.5- D_LCx_: 1.5- ɣ:159- α:90, (b) D_LM_:4.18- D_LAD_:3.5- D_LCx_: 3.5- ɣ:159- α:90, (c) D_LM_:4.18- D_LAD_:3.5- D_LCx_: 3.5- ɣ:112.75- α:130, (d) D_LM_:4.18- D_LAD_:3.5- D_LCx_: 3.5- ɣ:160- α:45, (e) D_LM_:4.18- D_LAD_:3.5- D_LCx_: 3.5- ɣ:112.75- α:90, (f) D_LM_:4.18- D_LAD_:3.5- D_LCx_: 3.5- ɣ:159- α:120.

**Ordering points**

**
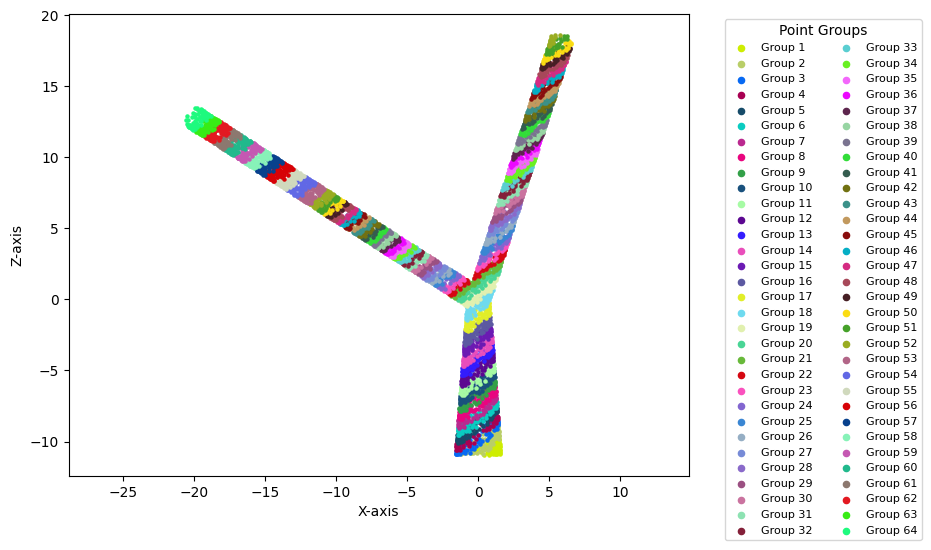
**

Figure S 3: A 2D representation of one of the point clouds after ordering. The 64 clusters, each containing 64 points, are shown in different colors to illustrate the effect of ordering.

Table S 3: Bifurcation Angulation geometry – Left Coronary Artery depicted in Figure 1.a.

| Study | ɣ (degree) | α (degree) |
| --- | --- | --- |
| Dong et al. (Dong et al., 2015) | 159 | 70 |
|  | 159 | 75 |
|  | 159 | 80 |
|  | 159 | 85 |
|  | 159 | 90 |
|  | 159 | 95 |
|  | 159 | 100 |
|  | 159 | 105 |
|  | 159 | 110 |
| Doutel et al. (Doutel et al., 2016) | 155 | 75 |
|  | 148.5 | 75.5 |
|  | 142.5 | 75 |
|  | 167 | 77.5 |
| Pinto et al. (Pinto and Campos, 2016) | 150 | 85 |
| Beier et al. (Beier et al., 2016) | 150 | 60 |
|  | 140 | 80 |
|  | 160 | 40 |
| Chaichana et al. (Chaichana et al., 2011) | 120 | 120 |
|  | 127.5 | 105 |
|  | 135 | 90 |
|  | 157.5 | 45 |
|  | 165 | 30 |
|  | 172.5 | 15 |
| Kamangar et al. (Kamangar et al., 2017) | 159 | 30 |
|  | 159 | 60 |
|  | 159 | 120 |
| Liu et al. (Li et al., 2021b) | 180 | 60 |
|  | 180 | 90 |
|  | 180 | 120 |

Table S 4: Arteries’ Diameters– Left Coronary Artery depicted in Figure 1.a.

| Study | D_LM_ (mm) | D_LAD_ (mm) | D_LCx_ (mm) |
| --- | --- | --- | --- |
| Dong et al. (Dong et al., 2015) | 4 | 3.4 | 3 |
| Doutel et al. (Doutel et al., 2016) | 4 | 3.5 | 2.8 |
| Dodge et al. (Dodge et al., 1992) | 4.5 | 3.6 | 3.4 |
| Kiviniemi et al. (Kiviniemi et al., 2004) | 4 | 3.4 | 2.9 |
| Ballesteros and Ramirez. (Ballesteros and Ramirez, 2008) | 3.6 | 3.4 | 3 |
| Dombe et al. (Dombe et al., 2012) | 3.3 | 3.19 | 2.94 |
| Pinto et al. (Pinto and Campos, 2016) | 4.12 | 3.5 | 3 |
| Beier et al. (Beier et al., 2016) | 2.18 | 3.11 | 2.64 |
|  | 3.59 | 2.53 | 3.08 |
|  | 3.71 | 3.41 | 2.81 |
|  | 4.78 | 4.41 | 4.80 |
| Chaichana et al. (Chaichana et al., 2011) | 3 | 2 | 1.5 |
| Kamangar et al. (Kamangar et al., 2017) | 3 | 2 | 1.5 |
| Bahrami et al. (Bahrami and Norouzi, 2018) | 4.5 | 3.9 | 4 |
| Govindaraju et al. (Govindaraju et al., 2016) | 3 | 2 | 1.5 |
| Jahromi et al. (Jahromi et al., 2019) | 4.5 | 3.6 | 3.4 |
| Rabbi et al. (Rabbi et al., 2020) | 3 | 1.5 | 2 |
| Malve et al. (Malvè et al., 2012) | 5 | 3.75 | 3.25 |

Table S 5: Comparison of Encoding and Decoding Sessions on Prediction Performance.

| Number of encoding/decoding sessions | Performance (MRE%) |
| --- | --- |
| 1 | 25.7 |
| 2 | 18.59 |
| 3 | 14.69 |
| 4 | 11.39 |

Table S 6: Effect of the Number of Convolutional Layers in the Final Encoding Section on Prediction Performance.

| Number of Convolutional layers in the last encoded section | Performance (MRE%) |
| --- | --- |
| 1 | 19.32 |
| 2 | 16.85 |
| 3 | 15.33 |
| 4 | 11.39 |
| 5 | 15.33 |

Table S 7: Impact of Skip Connections on Prediction Performance.

| Skip connection | Performance (MRE%) |
| --- | --- |
| Yes | 11.39 |
| No | 30.35 |

Table S 8: Impact of Kernel Size on Prediction Performance.

| Kernel Size | Performance (MRE%) |
| --- | --- |
| 2 | 20.14 |
| 3 | 11.39 |
| 4 | 13.41 |
| 5 | 15.44 |
| 6 | 23.01 |

**Point Net**

The PointNet model, previously used in two studies (Li et al., 2021b, 2021a), was also trained on this dataset. Despite having 206,221 trainable parameters, it yielded a mean relative error (MRE) of 24.04% on the test dataset.

**References:**

Bahrami, S., and Norouzi, M. (2018). A numerical study on hemodynamics in the left coronary bifurcation with normal and hypertension conditions. *Biomech. Model. Mechanobiol.* 17, 1785–1796. doi: 10.1007/s10237-018-1056-1

Ballesteros, L. E., and Ramirez, L. M. (2008). Morphological expression of the left coronary artery: A direct anatomical study. *Folia Morphol. (Warsz).* 67, 135–142.

Beier, S., Ormiston, J., Webster, M., Cater, J., Norris, S., Medrano-Gracia, P., et al. (2016). Impact of bifurcation angle and other anatomical characteristics on blood flow - A computational study of non-stented and stented coronary arteries. *J. Biomech.* 49, 1570–1582. doi: 10.1016/j.jbiomech.2016.03.038

Chaichana, T., Sun, Z., and Jewkes, J. (2011). Computation of hemodynamics in the left coronary artery with variable angulations. *J. Biomech.* 44, 1869–1878. doi: 10.1016/j.jbiomech.2011.04.033

Dodge, J. T., Brown, B. G., Bolson, E. L., and Dodge, H. T. (1992). Lumen diameter of normal human coronary arteries: Influence of age, sex, anatomic variation, and left ventricular hypertrophy or dilation. *Circulation* 86, 232–246. doi: 10.1161/01.CIR.86.1.232

Dombe, D., Anitha, T., Dombe, P., and Ambiye, M. (2012). Clinically relevant morphometric analysis of left coronary artery. *Int. J. Biol. Med. Res.* 3, 1327–1330. Available at: http://www.biomedscidirect.com/449/clinically_relevant_morphometric_analysis_of_left_coronary_artery/articles

Dong, J., Sun, Z., Inthavong, K., and Tu, J. (2015). Fluid–structure interaction analysis of the left coronary artery with variable angulation. *Comput. Methods Biomech. Biomed. Engin.* 18, 1500–1508. doi: 10.1080/10255842.2014.921682

Doutel, E., Pinto, S. I. S., Campos, J. B. L. M., and Miranda, J. M. (2016). Link between deviations from Murray’s Law and occurrence of low wall shear stress regions in the left coronary artery. *J. Theor. Biol.* 402, 89–99. doi: 10.1016/j.jtbi.2016.04.038

Govindaraju, K., Badruddin, I. A., Viswanathan, G. N., Kamangar, S., Salman Ahmed, N. J., and Al-Rashed, A. A. A. A. (2016). Influence of variable bifurcation angulation and outflow boundary conditions in 3D finite element modelling of left coronary artery on coronary diagnostic parameter. *Curr. Sci.* 111, 368–374. doi: 10.18520/cs/v111/i2/368-374

Jahromi, R., Pakravan, H. A., Saidi, M. S., and Firoozabadi, B. (2019). Primary stenosis progression versus secondary stenosis formation in the left coronary bifurcation: A mechanical point of view. *Biocybern. Biomed. Eng.* 39, 188–198. doi: 10.1016/j.bbe.2018.11.006

Kamangar, S., Badruddin, I. A., Badarudin, A., Nik-Ghazali, N., Govindaraju, K., Salman Ahmed, N. J., et al. (2017). Influence of stenosis on hemodynamic parameters in the realistic left coronary artery under hyperemic conditions. *Comput. Methods Biomech. Biomed. Engin.* 20, 365–372. doi: 10.1080/10255842.2016.1233402

Kiviniemi, T. O., Saraste, M., Koskenvuo, J. W., Airaksinen, K. E. J., Toikka, J. O., Saraste, A., et al. (2004). Coronary artery diameter can be assessed reliably with transthoracic echocardiography. *Am. J. Physiol. - Hear. Circ. Physiol.* 286, 1515–1520. doi: 10.1152/ajpheart.00819.2003

Li, G., Song, X., Wang, H., Liu, S., Ji, J., Guo, Y., et al. (2021a). Prediction of Cerebral Aneurysm Hemodynamics With Porous-Medium Models of Flow-Diverting Stents via Deep Learning. *Front. Physiol.* 12, 1–13. doi: 10.3389/fphys.2021.733444

Li, G., Wang, H., Zhang, M., Tupin, S., Qiao, A., Liu, Y., et al. (2021b). Prediction of 3D Cardiovascular hemodynamics before and after coronary artery bypass surgery via deep learning. *Commun. Biol.* 4, 1–12. doi: 10.1038/s42003-020-01638-1

Malvè, M., García, A., Ohayon, J., and Martínez, M. A. (2012). Unsteady blood flow and mass transfer of a human left coronary artery bifurcation: FSI vs. CFD. *Int. Commun. Heat Mass Transf.* 39, 745–751. doi: 10.1016/j.icheatmasstransfer.2012.04.009

Pinto, S. I. S., and Campos, J. B. L. M. (2016). Numerical study of wall shear stress-based descriptors in the human left coronary artery. *Comput. Methods Biomech. Biomed. Engin.* 19, 1443–1455. doi: 10.1080/10255842.2016.1149575

Rabbi, M. F., Laboni, F. S., and Arafat, M. T. (2020). Computational analysis of the coronary artery hemodynamics with different anatomical variations. *Informatics Med. Unlocked* 19, 100314. doi: 10.1016/j.imu.2020.100314
